# Molecular authentication of sika deer (*Cervus nippon*) based on allele-specific PCR

**DOI:** 10.1080/23802359.2019.1624205

**Published:** 2019-07-11

**Authors:** Feixia Hou, Jihai Gao

**Affiliations:** Pharmacy College, Chengdu University of Traditional Chinese Medicine, Chengdu, Sichuan, 611137, China

**Keywords:** Sika deer, molecular authentication, COI, allele-specific PCR

## Abstract

Sika deer (*Cervus nippon*) is listed as a tonic in many ancient Chinese pharmaceutical. Many adulterants of sika deer products have been found in Chinese medicinal materials markets, which led to detrimental impacts in clinical treatment. However, it is lack of the rapid and effective identification method for sika deer. This study amplified 574 bp fragment of mtDNA COI region of 19 samples from seven Cervidae species, and the relevant five sequences from reindeer (*Rangifer tarandus*) were downloaded from GenBank. It was found that there were two SNP loci for sika deer. Phylogenetic analysis indicated that individuals from sika deer clustered together. Based on SNP locus, one pair specific primer for allele-specific PCR identification of sika deer was designed, which could be used to rapidly and accurately identify sika deer.

Deer belongs to Cervidae which includes 10 genus and 17 species in China. The majority of these species are usually used in folk medicine to tonify Kidney Yang, benefit life essence and blood, and strengthen tendons and bones (Bi et al. 2016). Furthermore, deer anlter, called “Lurong” in China, is widely sold in many Asian countries such as Korea and China as a natural dietary supplement. However, the authoritative sources of Lurong only are the anlters of sika deer (*Cervus nippon*) and red deer (*Cervus elaphus* Linnaeus) according to Chinese Pharmacopoeia (Editorial Committee of China Pharmacopoeia 2015). Due to stiff price, many adulterants of Lurong have been found in the market.

Sika deer, which is listed as a tonic in many ancient Chinese pharmaceutical monographs, is famous and valuable animal in China and has been bred in captivity for deer antlers for the Chinese medicine market for many years. Although the number of wild population is in growth at present due to habitat restoration, sika deer is still listed in Category I of the List of Chinese State Key Protected Wild Animals. It is illegal to deal products of sika deer if not being permitted by the government. Moreover, according to the Wild Animal Conservation Law of China, punishment for smuggling different deer species is different. So authentication of sika deer is very necessary and significant. There were some literature that applied traditional identification methods including morphological (Chen et al. 1999), microscopic (Liu and Kang 2009), physical and chemical (Zhou et al. 2001; Wang et al. 2001; Mao et al. 2007) to identify products of sika deer. However, the accuracy of these approaches often relies on authenticators’ subjective experience and is more influenced by external factors. Therefore, it is necessary to find a convenient and reliable means for authenticating products of sika deer. Compared to traditional approaches, DNA-based technology authentication methods are more reliable, accurate, efficient, and stable. DNA barcoding based on mitochondrial cytochrome c oxidase subunit I (COI) is recognized as a standard tool for animal identification (Yan et al. 2013). Nevertheless, the technique, which needs large instruments and sequencing, is high cost and time-consuming. What is more, it relies on the accuracy of nucleotide sequence of recorded species in database such as NCBI.

Allele-specific PCR (AS-PCR), which no sequencing, is a simple, rapid, cheap, and reliable method for species identification. Since it was first used to identify black caviar (DeSalle and Birstein 1996), allele-specific PCR has been widely used in discrimination or authentication plants and animals (Ding et al. 2003; Liu and Guo, 2010; Wang et al. 2010; Jigden et al. 2010; Sun et al. 2012; Wang et al. 2012; Xu et al. 2013). In this study, AS-PCR based upon COI sequences that were amplified from 19 samples of seven Cervidae species was used to distinguish sika deer from its closely related species.

Nineteen hair samples of seven species from Cervidae: *C. nippon*, *C. elaphus*, *Axis porcinus*, *Dama dama*, *Elaphurus davidianus*, *Elaphodus cephalophus*, and *Rusa unicolor* were collected from Chengdu Zoo in Sichuan province of China. All specimens were deposited at the Ministry of Education Key Laboratory of Standardization of Chinese Herbal Medicine, Chengdu University of Traditional Chinese Medicine, Sichuan Province, China. Genomic DNA from the hair root of nineteen samples was extracted using PCR cycler method (Bai et al. 2012). COI regions were amplified using primers of COIF (5′-TTGGTGCCTG AGCAGGCATAGT-3′) and COIR (5′-GAGAACAAGTGTTGAT ATAGAAT-3′) (Zhang et al. 2011). PCR products were bidirectionally sequenced, and then bidirectional DNA sequences were assembled using ContigExpress software. Finally, 574 bp fragment of COI region of 19 samples from seven Cervidae species were obtained (accession no. KX377310-KX377326, KX388145, KX388146). The relevant five sequences from reindeer (*Rangifer tarandus*) (accession no. JF443386, JF443512, KJ205574, JF443413, and KY385852) were downloaded from GenBank. Overall, we analyzed 24 partial sequences of COI region from eight Cervidae species. The phylogenetic analysis showed that individuals of *C. nippon*, *C. elaphus*, *A. porcinus*, *D. dama*, *E. davidianus*, *E. cephalophus*, *R. unicolor*, and *R. tarandus* were separately clustered within their corresponding clades, and *C. nippon* distinctly differs from other seven Cervidae species (Figure 1).

**Figure 1.**
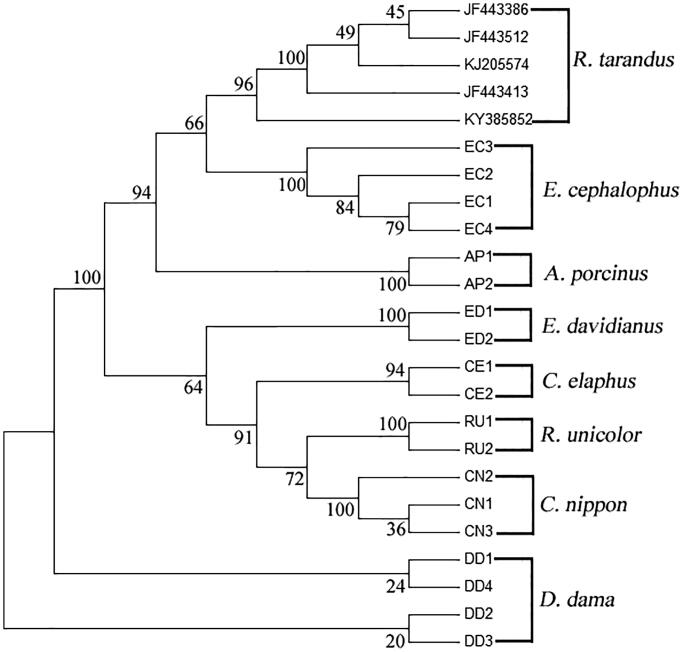
Phylogenetic analysis using NJ algorithm among eight Cervidae species based on COI sequences. The robustness was assessed by performing bootstrapping analysis with 1000 replicates.

There were two SNP loci specific to sika deer, which were located at nucleotide positions 480 and 505, respectively (Figure 2a). The bases of sika deer at sites 480 and 505 were C, whereas the other seven Cervidae species were T (Figure 2a). The 480-specific base C was selected to design the allele-specific downstream primer for sika deer. The site 480-specific nucleotide C was used as the first nucleotide at the 3' terminal of the downstream primer (Figure 2a). To improve the specificity of allele-specific amplification, additional nucleotide mismatches (G→T) was introduced at the antepenult base of downstream primer (Figure 2a). So, the downstream primer sequence was 5′-GGTTTCGGTCTGTTAATAGTATTTTG-3′ named CNR. Primer CNF was designed as the forward primer, and its nucleotide sequence was 5′-TATCGTAACCGCACATGCATT-3′. The expected band size of PCR products was 474 bp.

**Figure 2.**
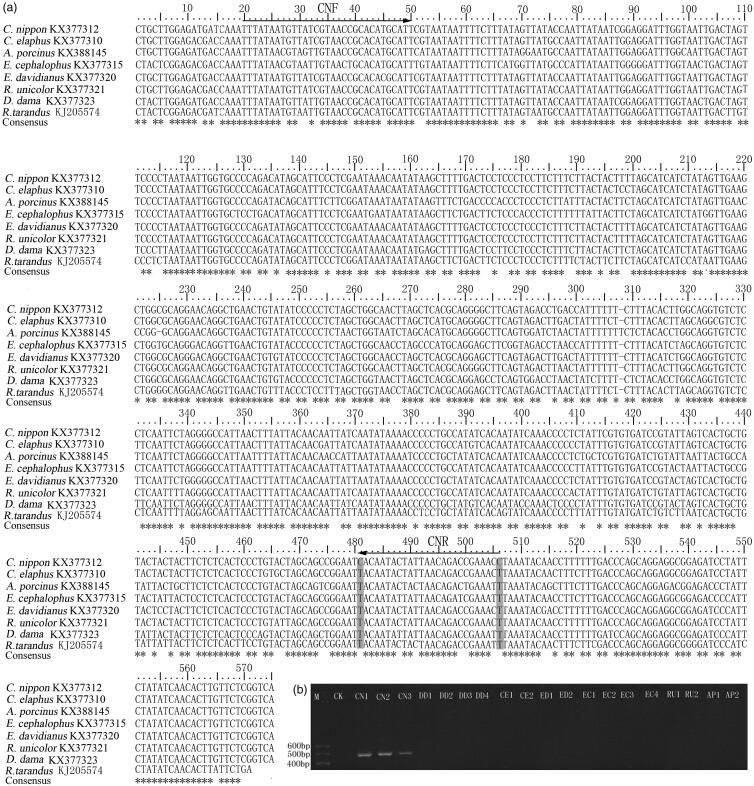
(a) Comparison of the COI sequences of eight Cervidae species (gray shadow: SNP loci of sika deer). (b) Products of allele-specific PCR using primers CNF/CNR.

Allele-specific PCR amplification was performed for the authenticating of sika deer using the specific primer. The PCR reactions were performed in 25 μl volumes comprising of 12.5 μl 2 × Taq master mix buffer (Tiangen, China), 1 pmol of each primer, and approximately120 ng of genomic DNA. The conditions of running PCR were set as the following: initial denaturation at 94 °C for 3 min, 35 cycles of denaturation (94 °C for 30 s), annealing (60 °C for 30 s), extension (72 °C for 30 s), and a final extension at 72 °C for 10 min. For verifying the species specificity of the primers for the identification of sika deer, the same PCR reaction constitutes and conditions were used for the other six species of Cervidae in this study. The result of electrophoresis showed that only the genomic DNA of sika deer could be successfully amplified and produced a single band of about 500 bp (Figure 2b), which indicated the pair primer had high specificity.

This study designed one pair specific primer of sika deer based on SNP loci of COI sequences. It was proved that the primer had strong specificity and could be used to rapidly and effectively identify the products related to sika deer.
